# Fractionated radiotherapy initiated at the early stage of bone metastasis is effective to prolong survival in mouse model

**DOI:** 10.1080/15384047.2025.2455756

**Published:** 2025-01-20

**Authors:** Yun Zhang, Zhunyi Gao, Ziwei Qi, Jiahe Xu, Jiao Xue, Lujie Xiong, Junhui Wang, Yuhui Huang, Songbing Qin

**Affiliations:** a Department of Radiotherapy, The First Affiliated Hospital of Soochow University, Suzhou, China; b Cyrus Tang Hematology Center, State Key Laboratory of Radiation Medicine and Prevention, Collaborative Innovation Center of Hematology, Soochow University, Suzhou, China; c Department of General Surgery, The Second Affiliated Hospital of Soochow University, Suzhou, China

**Keywords:** Fractionated radiotherapy, bone metastasis, secondary lung metastasis, early stage, immune cells

## Abstract

**Background and purpose:**

Bone metastasis is common for breast cancer and associated with poor prognosis. Currently, radiotherapy (RT) serves as the standard treatment for patients exhibiting symptoms of bone metastasis to alleviate pain. Whether earlier application of RT will better control bone metastasis remains unclear.

**Methods:**

We utilized a mouse model of breast cancer bone metastasis by intra-femoral injection of 4T1-luc breast tumor cells. The bone metastasis was treated by RT using various doses, timings, and modalities. Tumor growth was assessed through bioluminescence imaging, and lung metastases was quantified following lung tissue fixation. Flow cytometry was employed to analyze alterations in immune cell populations.

**Results:**

Single high-dose RT suppressed tumor growth of bone metastases, but caused severe side effects. Conversely, fractionated RT mitigated tumor growth in bone metastases with fewer adverse effects. Fractioned RT initiated at the early stage of bone metastasis effectively inhibited tumor growth in the bone, suppressed secondary lung metastases, and prolonged mouse survival. In line with the known pro- and anti-metastatic effects of neutrophils and T cells in breast cancer, respectively, earlier fractioned RT consistently decreased the proportions of neutrophils while increased the proportions of T cells in both the bone and the lung tissues.

**Conclusion:**

The data suggest that fractionated RT can inhibit the progression of early stage of bone metastasis and reduce secondary lung metastasis, leading to favorable outcomes. Therefore, these findings provide preclinical evidence to support the application of fractionated RT to treat patients with bone metastasis as earlier as possible.

## Introduction

Breast cancer is one of the most common types of malignant tumors worldwide, and systemic metastasis is the primary cause of death.^1^ Breast cancer frequently spreads to various organs, with bones being a predominant site. Despite advancements in primary breast cancer treatment, metastatic bone disease remains incurable.^2^ Median survival post bone metastasis in breast cancer varies between 2 to 3 years.^3,4^ Bone metastasis commonly results in skeletal morbidity, termed skeletal-related events (SREs).^5^ SREs diminish patients’ daily mobility, impair social functioning, and reduce their quality of life, incurring substantial medical costs.^6^ Palliative care is the primary approach to managing bone metastasis, a focusing on disease control and symptom relief.^7–9^

Radiotherapy (RT) is a standard pain relief treatment for bone metastasis patients.^10^ The response rates range from 70% to 80%, with one-third of patients achieving complete pain relief.^11^ RT targets tumor cells and impedes bone metastasis growth, providing much-needed pain relief. However, the treatment also has limitations. Recent preclinical studies have revealed that the bone microenvironment facilitated secondary metastasis of breast and prostate tumor cells to distant organs, including the bone, lung, liver, and brain.^12^ Advanced breast cancer frequently leads to multi-organ metastases, typically associated with a poor prognosis. RT for bone metastasis is generally administered after symptom onset, which may not effectively manage other organ metastasis resulting from the bone metastasis. Unfortunately, in such cases, patient survival cannot be improved.

Limited information is available regarding the application of RT for patients with asymptomatic bone metastasis. A recent clinical trial assessed the efficacy of RT in preventing SREs in asymptomatic patients.^13^ The trial findings suggested that prophylactic radiotherapy reduced the incidence of SREs and associated bone pain, indicating that early intervention may improve treatment outcomes. Nonetheless, the mechanisms driving these favorable results remain unclear.

In this study, we used a mouse model of breast cancer bone metastasis to investigate potential effects of RT on secondary lung metastasis and survival in mice. Specifically, we analyzed the efficacy of RT on local bone lesions, secondary lung metastasis, survival, and immune cells in multiple organs. Our results demonstrated that earlier fractionated RT mitigated bone metastasis and reduced lung metastasis, resulting in prolonged survival. This beneficial effect likely results from an increase in T cells and a reduction in neutrophils in the bone and the lungs. Therefore, RT could be an advantageous early-stage treatment option for bone metastasis in the clinical settings.

## Materials and methods

### Tumor cell culture

The 4T1 tumor cell line was purchased from the American Type Culture Collection (Manassas, VA, USA). The 4T1-luc cell line was purchased from Shanghai Jinyuan Biotechnology Co., Ltd (JY655). Tumor cells were cultured in RPMI-1640 medium containing 10% fetal bovine serum (FBS, Gibco) and 1% Penicillin-Streptomycin (Gibco) at 37°C in a humid incubator containing 5% CO_2_. Cell cultures were monitored for mycoplasma contamination, and only mycoplasma-negative cells were used in the experiments.

### Mouse model with breast cancer bone metastasis

Female BALB/c mice (6–8 weeks old) were purchased from the Beijing Vital River (Beijing, China) and Shanghai Laboratory Animal Center (Shanghai, China). All mice were housed in the specific-pathogen free (SPF) grade animal facility in Soochow University. 1 × 10^5^ 4T1-luc tumor cells were injected into the femur of mice by intra-femoral injection when mice were anesthetized. The tumor cells on the legs were measured by BLI before RT. BLI positive mice were then randomly divided into two groups: one for RT and the other as control. Then, tumor growth in the legs was monitored weekly with BLI. At the end of the experiment (usually three weeks after intra-femoral injection of tumor cells), the mice were euthanized with an overdose of pentobarbital sodium. All animal studies were approved by the Institutional Laboratory Animal Care and Utilization Committee of Soochow University (SUDA20161014A01). All experimental methods were performed in accordance with the Chinese Regulations for the Protection and Use of Animals.

### Irradiation

Tumor irradiation was conducted according to previous publications with minor modifications^14,15^: 6 Mev electron beams were delivered at a dose rate of 3 Gy/min using a Varian VitalBeam linear accelerator (Varian Medical System, Palo Alto, CA, USA) (25 cm × 25 cm light-limited cylinder, 100 cm source skin distance). The mice were anesthetized with intraperitoneal injections of pentobarbital sodium before irradiation. A lead mold with a 2 cm square radiation field was designed to specifically irradiate a tumor while protect the other area from irradiation (Fig S1a). The appropriate dose of radiotherapy was given within the appropriate range centered on the tumor site. To prevent hypothermia and death, the mice were placed on a warm electric blanket immediately after irradiation.

### Bioluminescence imaging

Bioluminescence imaging was performed weekly using an IVIS Lumina II. Mice were anesthetized and injected intraperitoneally with 150 μL (15 mg/mL) of D-luciferin. Mice were placed on the imaging platform of an IVIS Lumina II for imaging with continuous inhalation of isoflurane. After imaging, the condition of the mice was closely monitored to ensure that they were fully awake. The fluorescence of the legs was quantified by Living Image software.

### Lung tissue fixation and analysis

The mice were euthanized with intraperitoneal injections of 200 μL pentobarbital sodium (12.50 mg/mL) after termination of the experiment. The lungs of the mice were removed and placed in Bouin’s solution for 24 h for fixation. After being washed by ethanol, the lungs were placed on a clean background, and the number of metastatic nodules in both the front and back sides of the lung was independently counted under a stereo microscope and averaged by two individuals.

### Flow cytometric analysis

Peripheral blood, the bone marrow and the lung were collected and processed into single cell suspensions after the mice were euthanized. Rat anti-mouse CD16/CD32 (BD Pharmingen) antibody was diluted with flow buffer at 1:50, and 10 μL of the diluted CD16/CD32 antibody was added into the single-cell suspensions with two million cells. After staining, cells were washed with flow buffer (1% BSA, 0.1% NaN_3_ in PBS). The following fluorochrome-conjugated antibodies were used: PE-Cy7-CD8a, PE-CD4, APC-Cy7-CD25, FITC-Ly6G, BV421-CD45, BV510-CD11b, APC-Gr1, APC-Cy7-NK1.1, FITC-B220, PE-F4/80, AF700-CD4 (BioLegend). Add 5 μL 7-Amino-actinomycin D (7AAD) (eBioscience) to each tube of sample right before flow analysis. Flow cytometry data were acquired on a Gallios flow cytometer (Beckman) and analyzed with a Kaluza software (version 1.3).

### Statistical analysis

Statistical analyses were conducted by using Prism software (version 9, GraphPad). Experimental differences were tested using unpaired two-tailed Student’s *t*-test when comparing two independent groups and one-way ANOVA when comparing more than two groups. Survival curve was assessed by log rank test. All of the data were presented as the mean ± standard error of the mean (SEM). The results were considered statistically significant when *p* < 0.05.

## Results

### Breast tumor cells in the bone lead to secondary lung metastasis in mice

To investigate the effects of RT on bone metastases, we established a model of breast cancer bone metastasis through intra-femoral (IF) injection of 4T1-luc breast tumor cells into the bone marrow (Fig. S1b). All mice developed lung metastases within 3 weeks post-injection. To exclude the possibility that tumor cells spread to the lungs directly via blood vessels immediately after injection, we resuspended 4T1-luc cells in 50% Matrigel and injected the mixture into the bone. Matrigel uniquely solidifies at 37°C, thus preventing the rapid migration of tumor cells through the bloodstream to the lungs. There was no significant difference in the numbers of lung metastasis nodules between the control and the Matrigel mixture groups (Fig. S1c and d). These data suggested that tumor cells in the bone microenvironment contributed to secondary lung metastasis. The findings from our mouse model were consistent with previous report.^12^

### Radiotherapy effectively suppresses the tumor growth in the bone, yet single high-dose RT causes significant adverse effects

**Figure 1.**
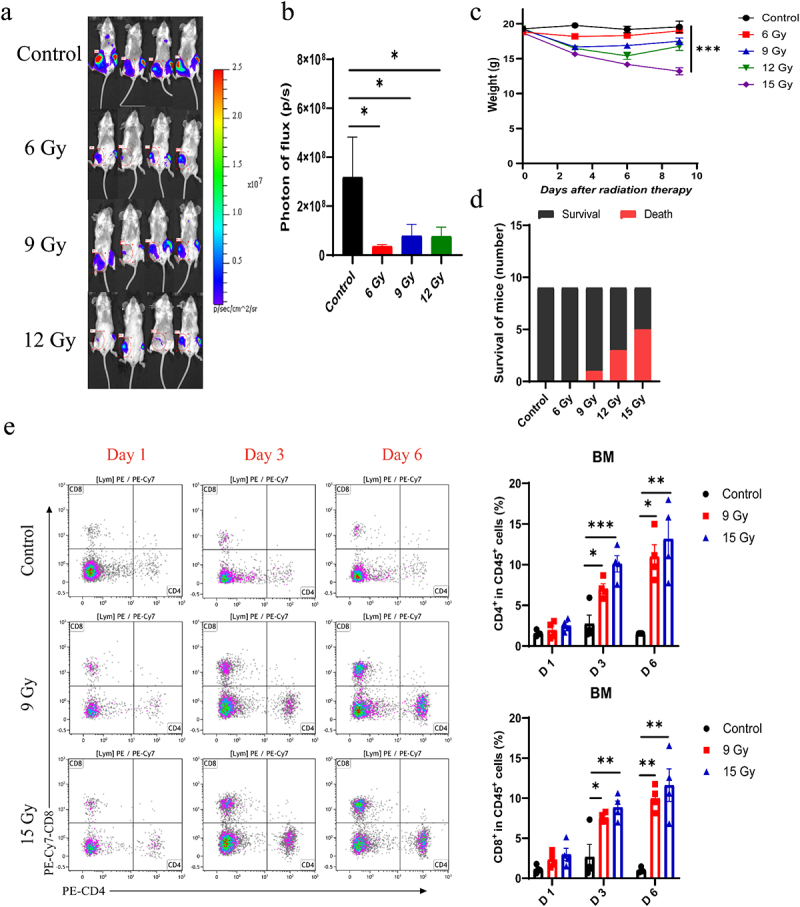
Radiotherapy inhibited tumor growth of bone metastasis, but single high-dose RT caused severe side effects. a, b. The bioluminescence images and quantification of tumors in the bone area of the mice injected with 4T1-luc cells on day 10 after RT (sRT was given 14 days post injection, *n* = 6). c. The weight curves of mice. Mice were treated with different doses of radiotherapy (sRT was given 8 days post injection). Weight was measured every three days after RT (*n* = 9). d. Mouse deaths were recorded during the experiment (*n* = 9). e. The proportions of CD4^+^ T and CD8^+^ T cells in bone marrow at days 1, 3, 6 after RT. The bone marrow was irradiated without inoculating 4T1-luc tumor cells (*n* = 4). The data are presented as means ± SEM. *P  < 0.05, **p <0.01, ***p <0.001.

To determine the optimal radiation dose for treating breast cancer bone metastasis, mice were exposed to various doses of ionizing radiation 14 days post-tumor cell inoculation. Compared with the control group, radiation doses ranging from 6 to 12 Gy significantly suppressed tumor growth in the bone area (Figure 1a,b). Despite similar levels of tumor growth inhibition across these doses, mice exposed to higher doses (12 and 15 Gy) exhibited significant weight loss and increased mortality (Figure 1c,d). These results highlight that RT can suppress bone metastasis, but single high-dose causes severe side effects.

### Femur irradiation primarily affects immune cells in the bone marrow

Generally, RT is a localized treatment that exerts a direct cytotoxic effect on tumor cells while concurrently altering the tumor microenvironment.^16^ Given the significance of the bone marrow as an immune organ, assessing the effects of RT on the immune system is essential. Therefore, we analyzed immune cell populations in the bone marrow, peripheral blood, and the lungs at different time points after RT. According to the flow cytometry data, there was an increase in the percentages of CD4^+^ and CD8^+^ T cells in the bone marrow on days 3 and 6 post-RT (Figure 1e). Neutrophils initially rose on day 1 post-RT, then diminished by days 3 and 6 (Fig. S2a). Again, RT at 9 Gy and 15 Gy exhibited comparable effects on immune cells in the bone marrow. The impacts of RT on immune cell populations in the peripheral blood and the lungs were marginal (Fig. S2b and c). These data suggested that the impacts of RT on immune cells were predominantly localized to the irradiated site.

### Fractionated RT inhibits tumor growth in the bone without affecting body weight, but increases lung metastasis

**Figure 2.**
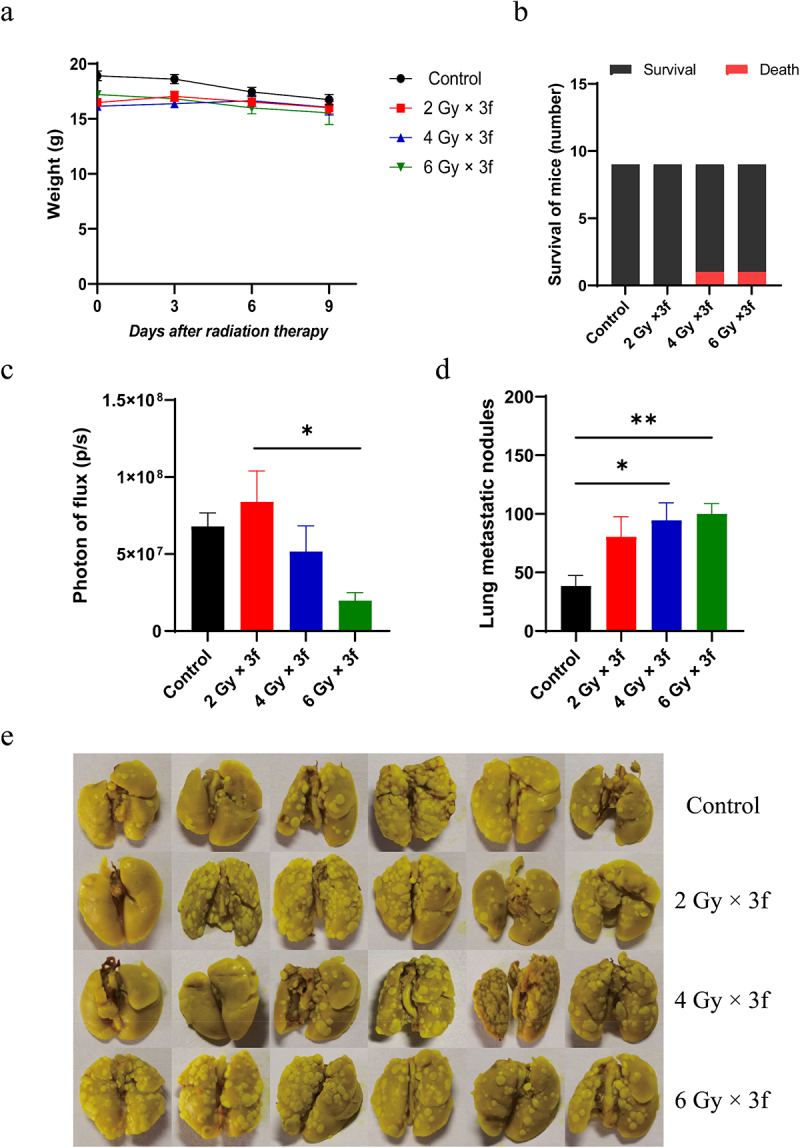
Fractionated RT on day 6 inhibited tumor growth of the bone metastasis with fewer side effects, but facilitated lung metastasis. a. The weight curves. The legs were treated with different doses of radiotherapy starting on day 6 after 4T1-luc tumor cells inoculation (*n* = 9). b. Mouse deaths were recorded during the experiment (*n* = 9). c. The leg tumor was quantified by bioluminescence imaging system on day 10 after RT in mice inoculating 4T1-luc cells. d, e. The quantification and representative images of lung metastasis in mice on day 10 after RT. The data are presented as means ± SEM. *P  < 0.05, **P  < 0.01.

Considering the adverse effects of a single high-dose of RT, we explored fractionated RT (fRT). Indeed, fRT did not cause significant weight loss in mice compared to control mice (Figure 2a). fRT at 6 Gy × 3f initiated on day 6 post-tumor cell injection showed a tendency to inhibit tumor growth in the bone area in mice. Unfortunately, fRT initiated on day 6 post-tumor cell injection increased the number of lung metastatic nodules in mice compared with the control group (Figure 2c-e). These results collectively showed that fractionated RT could inhibit tumor growth of bone metastasis with fewer side effects (Figure 2a-b), although it increased the risk of lung metastasis.

### Early fractionated RT is more effective in treating breast tumor bone metastasis

**Figure 3.**
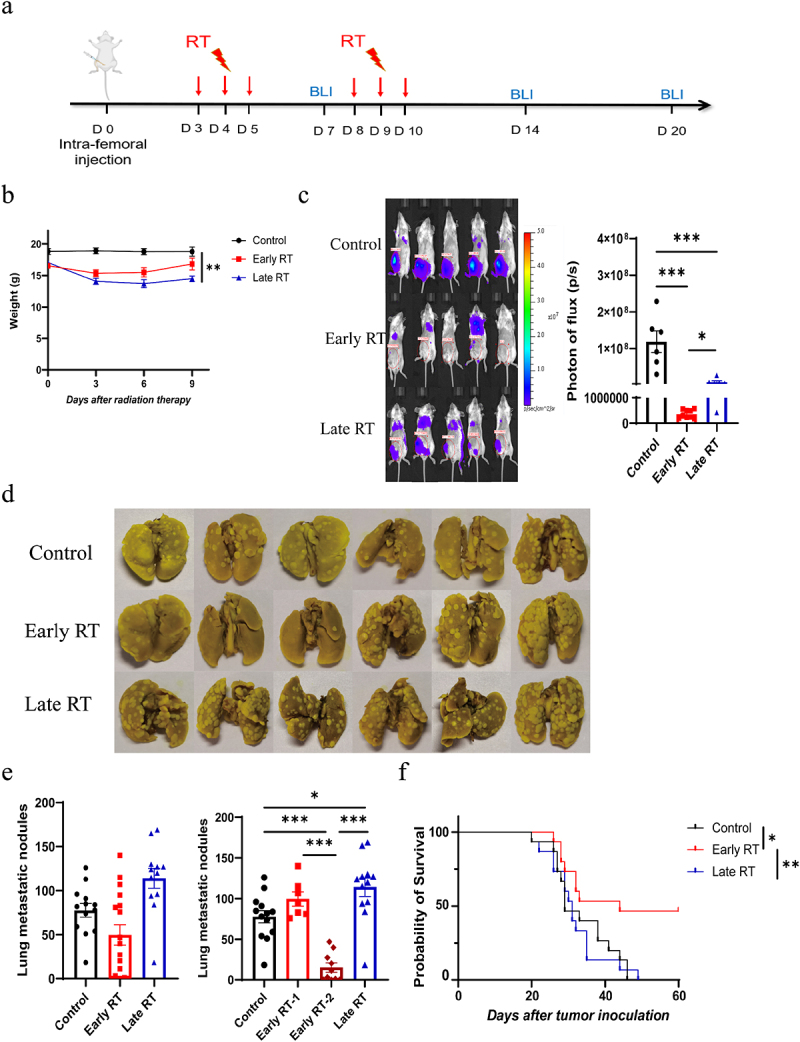
Early fractionated RT more effectively inhibited tumor growth of the bone metastasis and prolonged mouse survival compared with late fractionated RT. a. Experimental design: BALB/c mice were inoculated with 1 × 10^5^ 4T1-luc cells by intra-femoral injection. The mice were treated with radiation to the legs starting on day 3 (early stage) or day 8 (late stage) after 4T1-luc cell inoculation. When experiments were terminated, lung tissues were harvested for metastasis analysis. Tumor growth was monitored weekly by BLI (*n* ≥ 8). b. The weight curves. c. The bioluminescence images and quantification of leg tumors in mice. d. The representative images of lung metastasis in mice. e. The quantification of lung metastasis in mice. Left: early RT as a whole group; right: early RT was separated as two groups based on the mean number of nodules (early RT-1: the number of nodules not decreased, early RT-2: the number of nodules decreased). f. The survival of mice inoculated with 4T1-luc tumor cells received either early or late fractionated RT (*n* ≥ 14). The data are presented as means ± SEM. *p <0.05, **p <0.01, ***p <0.001.

Since fRT (6 Gy × 3f) on day 6 after tumor cells injection increased lung metastasis, we turned to compare the efficacy of early and late fRT which were initiated on day 3 and 8 after tumor cell inoculation, respectively (Figure 3a). The results revealed that mice in the late fRT group exhibited a significantly greater reduction in weight compared to those in the early fRT group (Figure 3b). fRT reduced tumor growth in bone metastases compared to the control group, with early fRT proving more effective (Figure 3c). Moreover, early fRT significantly reduced lung metastases and extended survival than those in the control group. However, there was no significant difference in lung metastasis and mouse survival between late fRT group and the control group (Figure 3d-f). These data suggest that fractionated RT initiated earlier is more effective in treating mouse bone metastasis.

### Early fractionated RT continuously elevates the proportions of T cells while reducing neutrophils

**Figure 4.**
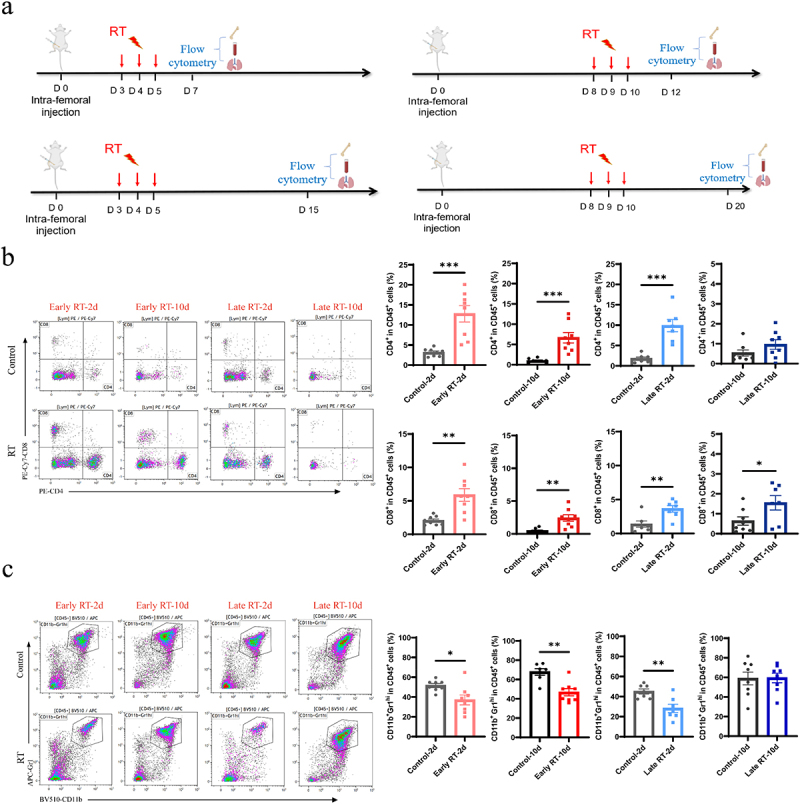
Early fractionated RT led to an increase in CD4^+^ and CD8^+^ T cells, while simultaneously decreasing neutrophils in the bone marrow on day 10 post-RT. a. Experimental design: the bone marrow, peripheral blood and the lung samples were harvested on days 2, 10 after early and late radiotherapy. Immune cells were analyzed by flow cytometry (*n* ≥ 8). b. The proportions of CD4^+^ and CD8^+^ T cells in the bone marrow. c. The proportions of neutrophils in the bone marrow. In our experiments, control-2d represented the control group on day 2 following radiotherapy. The days after tumor inoculation were identical for both groups, with the experimental group receiving radiotherapy and the control group not receiving any radiotherapy. The data are presented as means ± SEM. *P  < 0.05, **p < 0.01, ***p < 0.001.

It is widely known that neutrophils promote breast tumor lung metastasis while T effectors suppress it.^17–19^ We hypothesized that fRT may alter immune cells to facilitate secondary lung metastasis. We then analyzed immune cells in the bone marrow on days 2 and 10 post-early and late fRT (Figure 4a). Flow cytometry data revealed that, compared with control, fRT elevated the proportions of T cells in the bone marrow on day 2 after fRT. On day 10 post-RT, the early fRT group showed a significant increase in T cell proportions. (Figure 4b).

Concurrently, the proportion of neutrophils (CD11b^+^Gr1^hi^) was lower in fRT groups compared to controls on day 2 after fRT. This reduction persisted on day 10 after early fRT. However, there was no significant difference between the late fRT and the control group on day 10 after fRT (Figure 4c).

**Figure 5.**
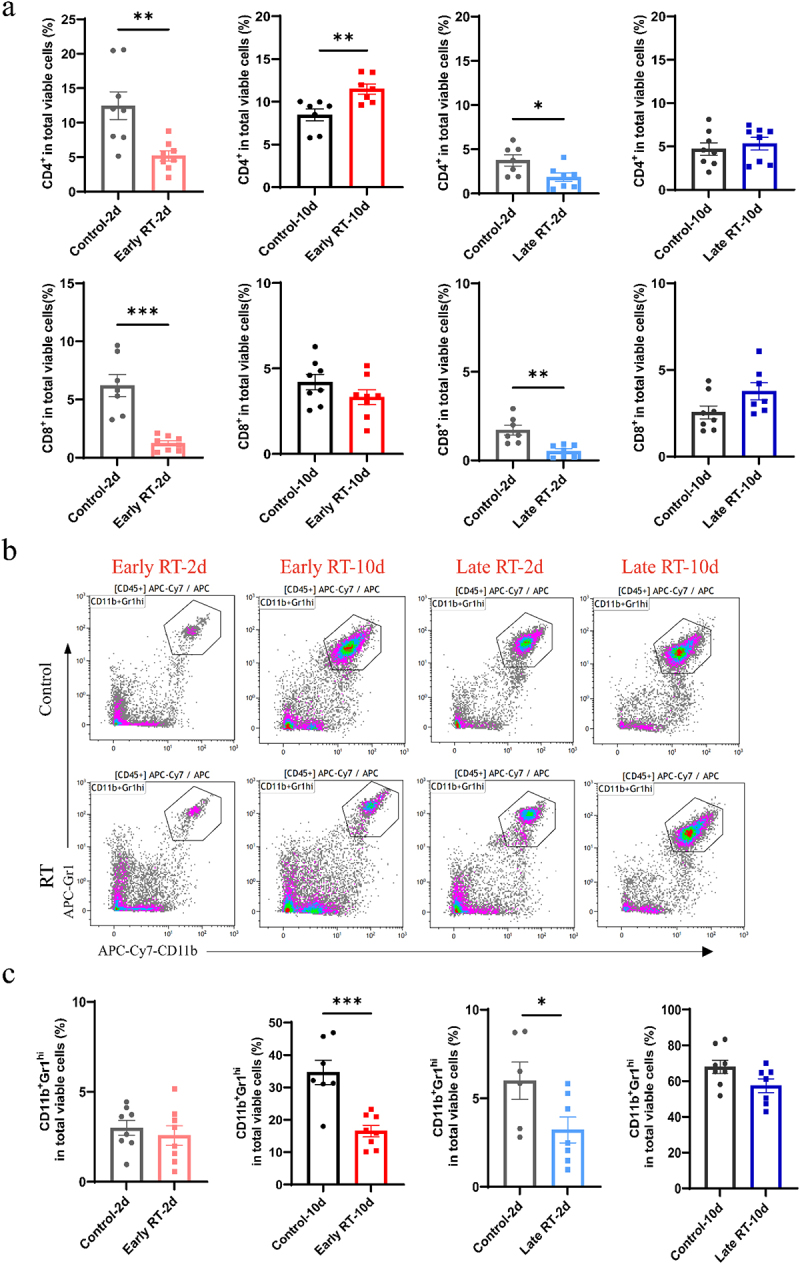
Early fractionated RT increased the rate of CD4^+^/CD8^+^ and decreased neutrophils in the lung on day 10 post-RT. a. The proportions of CD4^+^ and CD8^+^ T cells in the lungs. b, c. The proportions of neutrophils in the lungs. The data are presented as means ± SEM. *p <0.05, **p <0.01, ***p < 0.001.

We further analyzed immune cells in the peripheral blood (Fig. S3 and 4) and the lungs (Figure 5). Early fRT increased CD4^+^ T cell proportions in the lungs on day 10 compared to controls. Concurrently, neutrophil proportions in the lungs decreased on day 10 post-early fRT (Figure 5). However, the late fRT group did not cause such changes (Figure 5).

Taken together, these data indicated that early fractionated RT, but not late fractionated RT, can persistently increase the proportions of T cells and decrease neutrophils. CD4^+^ and CD8^+^ T cells possess the potential to exert an anti-tumor effect on tumor metastasis. Additionally, early RT reduced neutrophils in the long term which may prevent the formation of a pre-metastatic niche, thereby reducing lung metastasis.

## Discussion

Metastasis accounts for over 90% of cancer-related deaths.^20^ Breast cancer bone metastasis severely impacts patient prognosis.^6^ The primary objective of current treatments for bone metastasis is to mitigate symptoms. RT is one of the primary modalities for pain relief.^8,10^ However, bone metastasis can lead to secondary metastases in other organs, aggravating the condition.^12^ Early intervention in bone metastasis may improve survival. In this study, we demonstrated that fractionated RT conducted at the early stage of bone metastasis significantly inhibited secondary lung metastasis and extended survival in mouse model. These findings may help to upgrade treatment modality for breast cancer patients with bone metastasis.

RT relies on ionizing radiation to target tumor cells, causing DNA damage and potentially leading to cell death.^21^ While commonly used to treat cancer, it can cause adverse effects that significantly impair patient quality of life.^22^ In our study, mice receiving a high single dose of irradiation exhibited severe side effects. Conversely, fRT resulted in fewer side effects, making it a better candidate for further research. Therefore, balancing therapeutic benefits and adverse effects is essential for customizing treatments to individual needs.

Without compelling evidence, preventive and conventional RT for asymptomatic, uncomplicated bone metastasis is not typically recommended in clinical practice.^23^ However, a recent clinical study showed that preventive RT decreased the odds of subsequent SREs in asymptomatic high-risk patients with bone metastasis,^13^ highlighting its benefits. Early intervention is crucial as these tumors may spread to other organs,^12^ with timely treatment reducing proliferation and dissemination risks. We conducted several experiments to determine the optimal timing of RT to ensure effective early intervention. Our results revealed that lung metastases in mice increased when RT commenced after day 6 of tumor inoculation, suggesting that tumor cells had already migrated to the lungs in the early stages of the disease. Late RT failed to control lung metastasis if already spread to lungs, though it inhibited leg tumors. Therefore, we rescheduled the RT appointment to day 3 following tumor inoculation. Lung metastasis was suppressed in some mice at this time. These findings suggested that the timing of RT was crucial in treating bone metastasis, and early intervention may provide a more practical approach. These encouraging results could lead to novel clinical approaches for treating bone metastasis.

Metastasis is organ-specific. Breast cancer typically metastasizes to the bone, lung, liver, and brain.^24^ Tumor colonization, meanwhile, requires a suitable pre-metastatic niche. Primary tumors secrete soluble factors like cytokines, chemokines, and metabolites, inducing an immunosuppressive microenvironment in distant organs.^25^ These mainly include myeloid-derived suppressor cells (MDSC) such as myeloid cells, tumor-associated macrophages (TAM), tumor-associated neutrophils, and Tregs.^26^ Multiple studies have demonstrated the prominence of neutrophils in tumor metastasis.^19,27–29^ Our data revealed a reduction in bone marrow neutrophils on day 2 following RT. This decrease can be attributed to the direct cytotoxic effect of RT. On day 10 after early fRT, neutrophils levels in the bone marrow and lungs remained reduced. However, this was not observed after late fRT. Tumor secreted many myeloid suppressor cells, such as neutrophils. Fewer neutrophils were secreted when bone metastasis was suppressed after early fRT. Late fRT inhibited bone metastasis growth, though less effectively than early fRT. However, there was no significant difference in bone marrow neutrophils levels between late fRT and the control groups on day 10 post-RT. It appeared that the changes observed in the neutrophils were not only caused by tumors.

Normally, mature neutrophils are released from the bone marrow into the bloodstream. Like metastatic tumor cells, neutrophils can exit the bloodstream and migrate to various tissues. During acute infection and inflammation, many neutrophils migrate to the affected tissues.^30,31^ The IL-17/G-CSF axis regulates neutrophil production, mobilization, and elimination.^17^ Early fRT, rather than late fRT, might modulate the expression of neutrophil mobilizing and attracting chemokines.^32^ Specifically, it has been reported that neutrophils attract chemotactic signals such as S100A8/A9, CXCL1/2, and Lin28b to the pre-metastatic lungs, ultimately promoting metastasis. Furthermore, CXCL12 can be recruited to the pre-metastatic liver.^33–35^ Following early fRT, lung tissues showed reduced neutrophil levels and failed to attract these chemokines. Consequently, the lungs became inhospitable to tumor growth, leading to fewer secondary lung metastases in some mice. Additionally, there existed a crosstalk between the lung and bone. Osteoblasts have been reported to promote lung cancer by generating a subset of tumor-infiltrating SiglecF^high^ neutrophils.^36,37^ This process may be modulated by RT. These mechanisms might explain how early fRT inhibited lung metastasis in some mice. Further research is needed to clarify the precise mechanism.

CD4^+^ and CD8^+^ T cells are crucial in the immune response against tumors.^38^ Our data showed that the proportion of T cells in the bone marrow and CD4^+^ T cells in the lungs remained elevated on day 10 following early fRT. However, this outcome was not observed in the late fRT group. RT directly increased lymphocyte levels, and the reduction of myeloid cells also contributed to this increase.^39–41^ Therefore, we speculated that early fRT could suppress secondary lung metastasis originating from bone metastasis by modulating T cells in the microenvironment.

Our study demonstrated that early fRT significantly inhibited leg metastasis and reduced secondary lung metastasis, thereby extending survival in some mice. These findings suggested that early RT may be an effective strategy for managing bone metastasis. Our research indicated that alterations in the microenvironment of T cells and neutrophils could be intricately connected with this matter. To our knowledge, this is the first preclinical study investigating the effects of radiotherapy on early bone metastasis. Our data will provide fresh perspectives and a theoretical basis for optimizing clinical approaches toward managing bone metastasis. By sharing these findings, we hope to help improve treatment options for breast cancer patients with bone metastasis.

Although bone metastasis frequently occurs in breast, prostate, and lung cancers, our model was limited to breast cancer. Future studies will extend to other cancer types to enhance our understanding. Moreover, the bone metastasis model we utilized was not a spontaneous metastasis model, which presents certain limitations. Additional experiments are necessary to precisely determine how early fRT inhibits secondary metastasis in mice.

## Supplementary Material

Supplementary material_clean.docx

## Data Availability

All data generated during this study are included in the figures and supplementary information.
