# Supplementary material for: Fractionated radiotherapy initiated at the early stage of bone metastasis is effective to prolong survival in mouse model
Source: Cancer Biol Ther. 2025 Jan 20;26(1):2455756. doi: 10.1080/15384047.2025.2455756 (PMC12724103; doi:10.1080/15384047.2025.2455756)
Supplement: Supplementary material_clean.docx [file KCBT_A_2455756_SM3505.docx]

**Supplementary figure legends**

**Fig S1. Our lead mold for radiotherapy and breast cancer bone metastasis mouse model. a.** A lead mold with a 2 cm square radiation field. **b.** The diagram of intra-femoral (IF) injection. **c, d.** The quantification and representative images of lung metastasis after 3 weeks of the injection of 4T1-luc breast tumor cells into the bone (n=5). **e.** The quantification of lung metastasis in mice on day 10 after RT (n=9).

The data are presented as means ± SEM. ns, no significant difference.

**Fig S2. RT show little effects on immune cell populations in blood and the lung of naïve mice. a.** The proportions of neutrophils in bone marrow on days 1,3,6 after RT. The bone marrow was irradiated without inoculating 4T1-luc tumor cells (n=4). **b.** The proportions of CD4^+^ T cells, CD8^+^ T cells, and neutrophils in blood on days 1,3,6 after RT. The bone marrow was irradiated without inoculating 4T1-luc tumor cells (n=4). **c.** The proportions of CD4^+^ T cells, CD8^+^ T cells, and neutrophils in the lung on days 1,3,6 after RT. The bone marrow was irradiated without inoculating 4T1-luc tumor cells (n=4).

The data are presented as means ± SEM. * *P* < 0.05, ** *P* < 0.01, *** *P* < 0.001.

**Fig S3. The proportions of CD4^+^ and CD8^+^ T cells in blood of mice after RT.** The mice with RT were prepared as described in Fig. 4A. The data are presented as means ± SEM. * *P* < 0.05, ** *P* < 0.01.

**Fig S4. The proportions of neutrophils in blood of mice after RT.** The mice with RT were prepared as described in Fig. 4A. The data are presented as means ± SEM. * *P* < 0.05, *** *P* < 0.001.

**Fig S1**

**
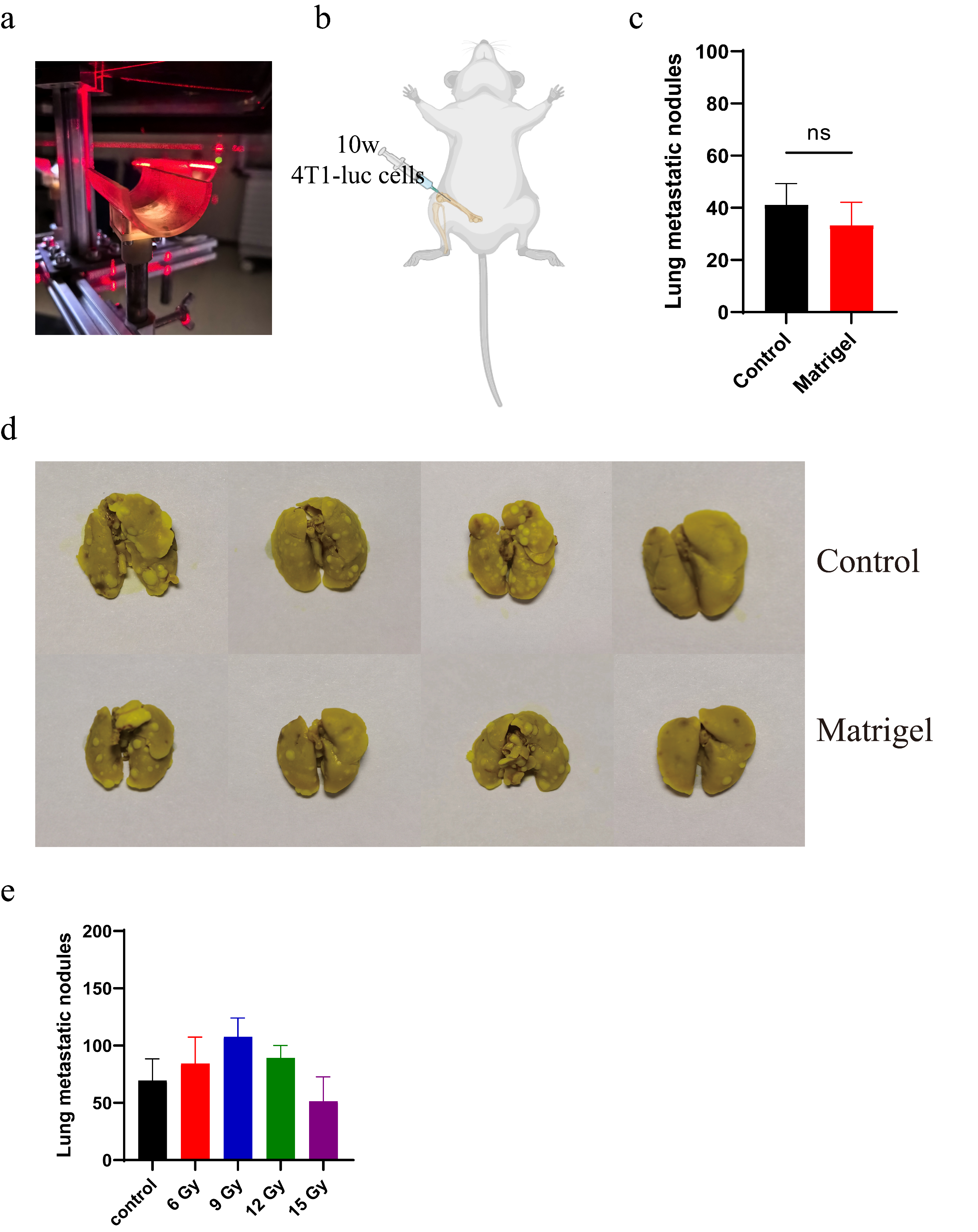
**

**Fig S2**

**
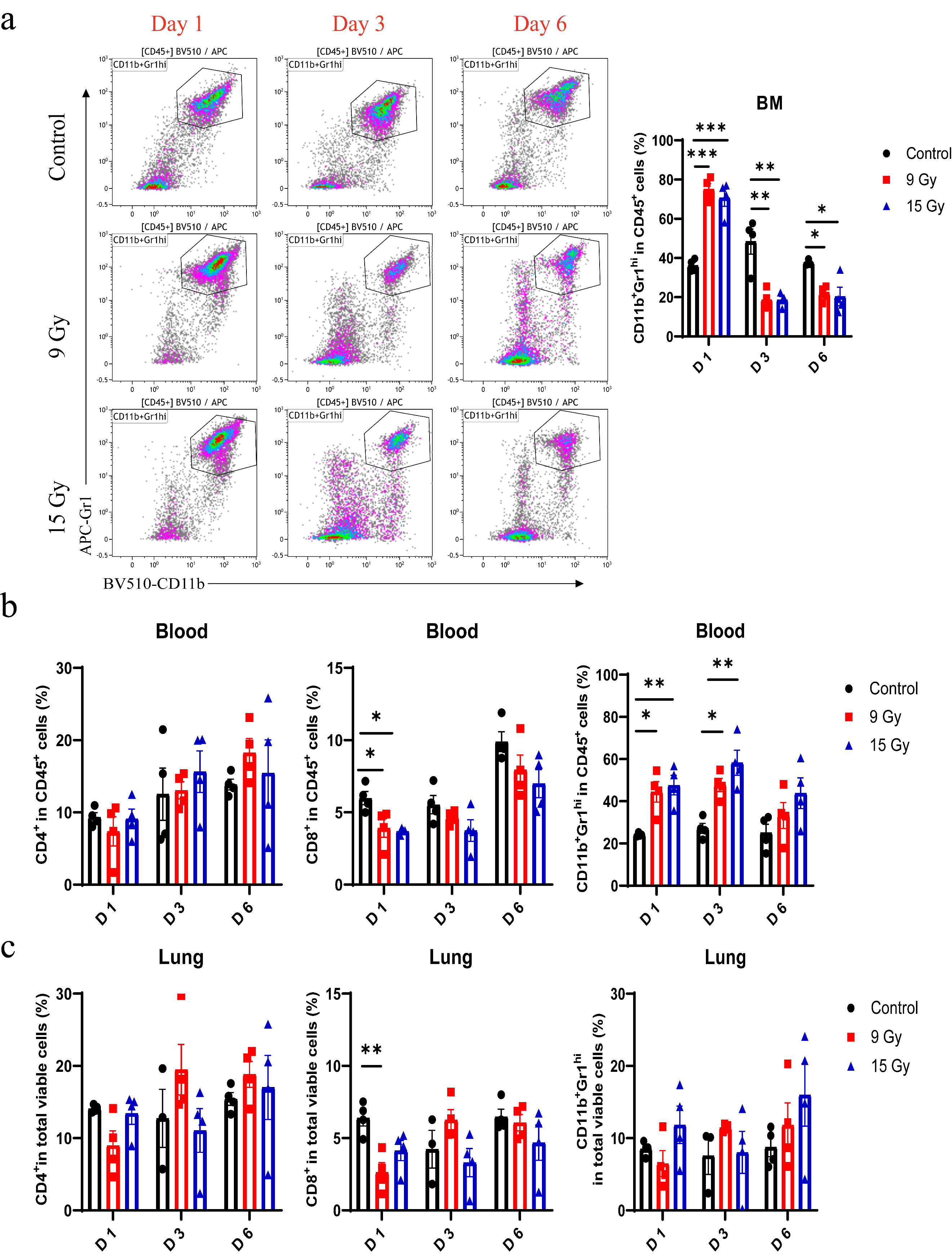
**

**Fig S3**

**
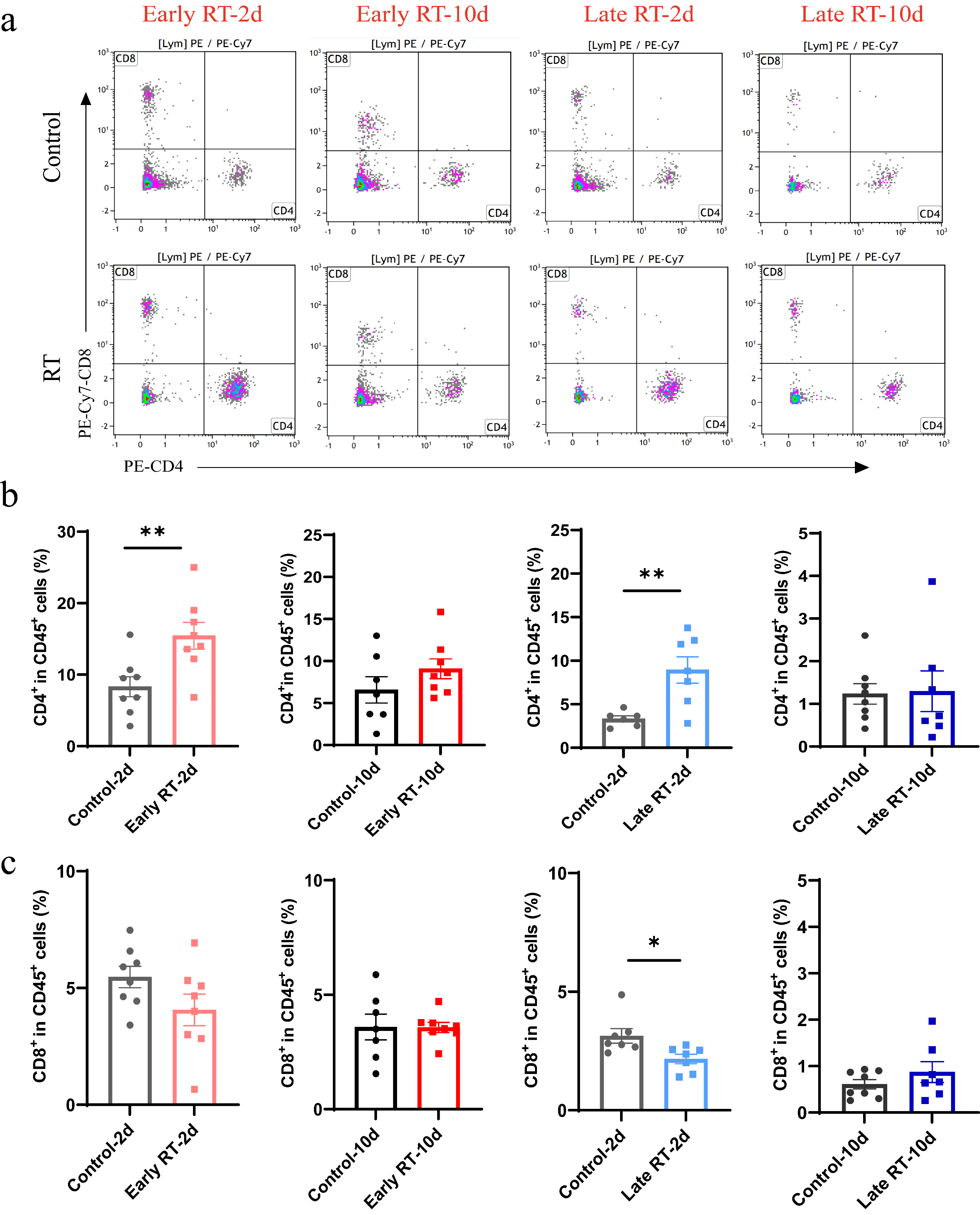
**

**Fig S4**

**
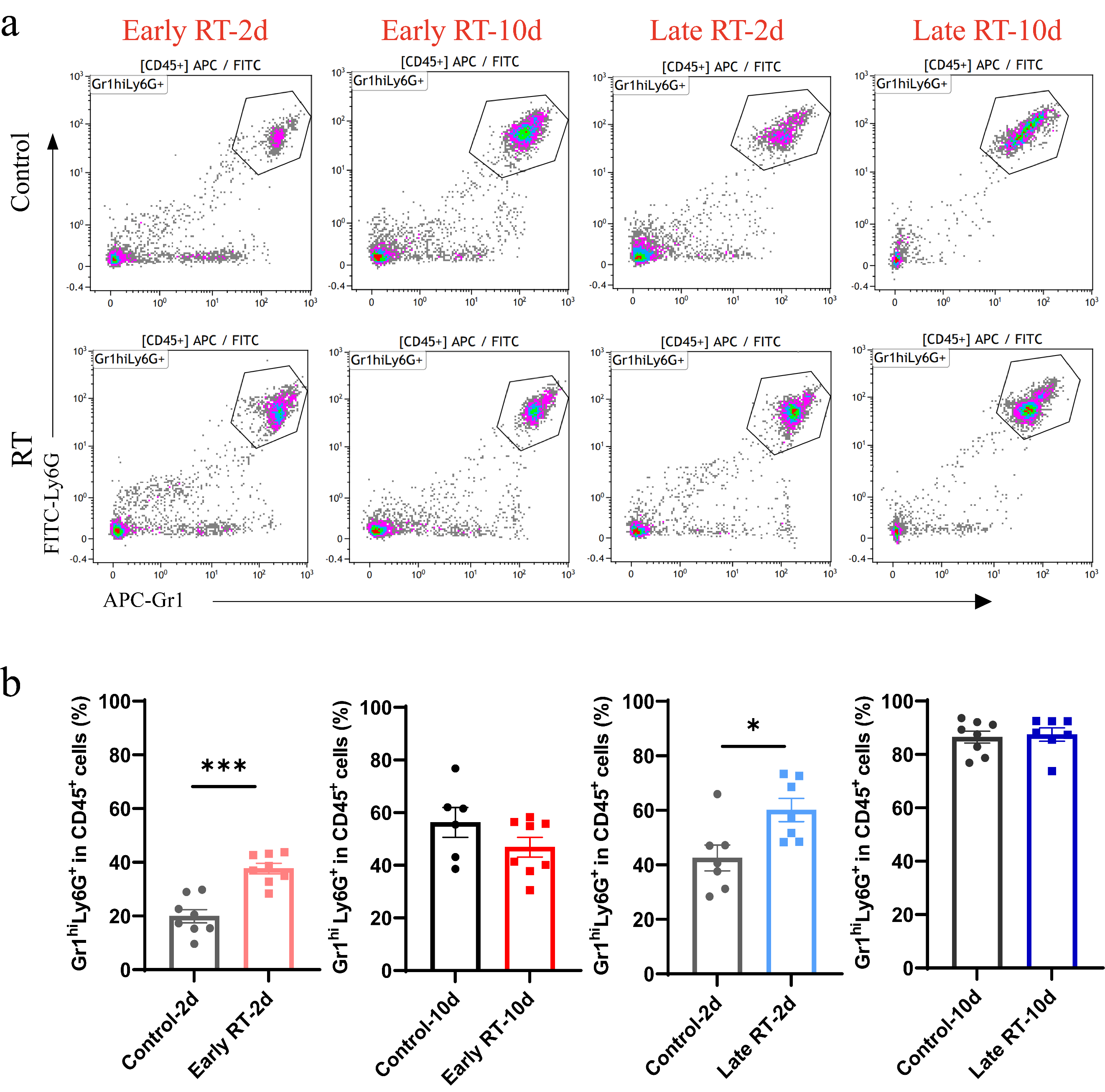
**
